# Microbes in mass extinction: an accomplice or a savior?

**DOI:** 10.1093/nsr/nwad291

**Published:** 2023-11-21

**Authors:** Genming Luo, Deng Liu, Hao Yang

**Affiliations:** State Key Laboratory of Geobiology and Environmental Geology, China University of Geosciences (Wuhan), China; State Key Laboratory of Geobiology and Environmental Geology, China University of Geosciences (Wuhan), China; State Key Laboratory of Geobiology and Environmental Geology, China University of Geosciences (Wuhan), China

## Abstract

The invisible microbes are the main components of the biosphere and proliferated in many mass extinctions of animals. Whether the proliferation of microbes was an accomplice or a savior of the mass extinction remains uncertain. Future work has to quantify the dual effects of microbes on the environment.

Microbes include various types of organisms that are invisible to the naked eye, e.g. bacteria, archaea and microeukaryotes. Although the precise number remains largely unknown, microbes are recognized as the unseen majority of our biosphere [1]. As a group of organisms classified by body size, microbes cover a considerable spectrum of metabolisms, including oxygenic photosynthesis, denitrification, sulphate reduction, methanogenesis and others. Therefore, microbes are supposed to be the engines that drive Earth's biogeochemical cycles [2], whose evolutionary history will be one of the 12 priority science questions of Earth science over the next 10 years [3]. In the ∼3.8-billion-year-long history of life, microbes were the only organisms of the biosphere in the first >80% of that history and played a substantial role in shaping the habitability of our Earth. Much attention has been paid to the evolution of macroorganisms (animals and plants) since the Phanerozoic, whereas microbes, the once chief actors on this planet, have been insensibly omitted. Intriguingly, it has been noted that the multiple mass extinctions of animals in the Phanerozoic were accompanied by the flourishing of microbes [4,5]. Are these microbes the accomplice or the savior when it comes to the mass extinction of animals?

**Microbes in mass extinction.** It is well-known that the evolutionary history of metazoans was punctuated by the five mass extinctions, known as the Big 5, occurring at the end-Ordovician (Hirnantian), Late Devonian (Frasnian-Famennian transition, F-F), Permian-Triassic transition (P-Tr), end-Triassic and end-Cretaceous. Although quantitative data are scarce, multiple geological and geochemical records reveal that extinctions of marine faunas were frequently accompanied by the proliferation of microbes.

The most prominent indicator of the proliferation of microbes is the wide distribution of microbialites directly above the mass extinction levels (Fig. 1 and Supplementary Fig. S1). Microbialites

are the primary components of the Precambrian sedimentary strata, and their abundance has decreased distinctly since the diversification of metazoans in the Ordovician [4]. However, the abundance and diversity of microbialites resurged following mass extinctions such as the F-F and the P-Tr mass extinctions. For example, various types of microbialites, including stromatolites, thrombolites and microbially induced sedimentary structures (MISSs), have been widely observed around the margins of the Paleo-Tethys, Neo-Tethys and the Panthalassa oceans after the P-Tr mass extinction (Fig. 1). Although the mechanism triggering the precipitation of microbialites remains elusive, it is a consensus that the primary constructors of these microbialites were microbes, which might have been opportunistic generalists due to the relaxation of ecological constraints. By using modern microbial mats as an analog, it is likely that the predominant microbes in deep-time microbialites could have been cyanobacteria.

**Figure 1.**
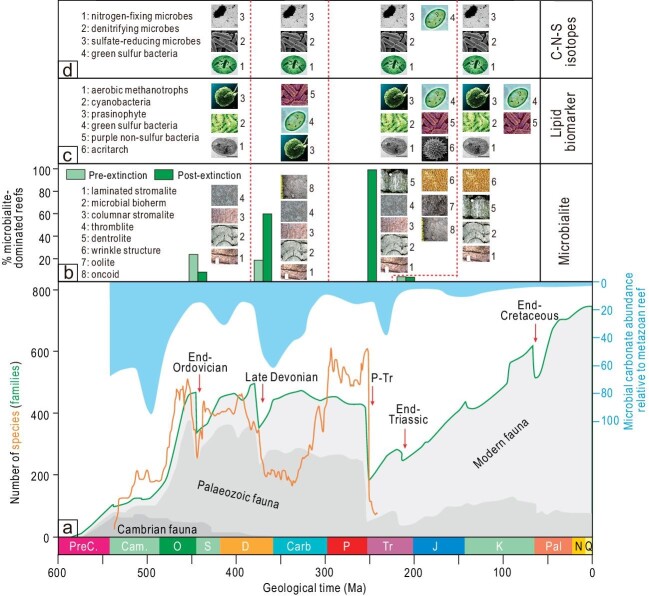
(a–d) Temporal variations of species and family diversity, relative abundance of microbialites, and primary records of the proliferation of microbes in the Phanerozoic (see the Supplementary Data online for a detailed description).

Lipid biomarker investigations also show the proliferation of microbes accompanying mass extinctions of metazoans. Since microbes are tiny and rarely preserved as fossils, they are usually absent in conventional paleontological studies. Lipids, a principal component of cell membranes that can be well preserved in sedimentary rocks, provide opportunities for understanding microbes in deep time. In the past two decades, based on lipid biomarker records, multiple types of microbes have been observed to have flourished after mass extinctions. For example, the proliferations of cyanobacteria, green sulfur bacteria, aerobic methanotrophs and acritarchs have been found after the Hirnantian, F-F, P-Tr and the end Triassic mass extinctions (Fig. 1).

The proliferation of microbes accompanying mass extinction has also been inferred by carbon, nitrogen and sulfur isotopes. It is well known that microbial metabolisms generally cause substantial isotope fractionation, with light isotopes typically enriched in metabolic products. For example, significant nitrogen isotope fractionation occurs during denitrification, and thus a high δ^15^ N value characterizes the residual nitrate. Carbon, nitrogen and sulfur isotopes have revealed the proliferation of nitrogen-fixation microbes, denitrifying microbes, sulfate-reducing microbes and possibly sulfur-disproportionating microbes during mass extinctions (Fig. 1).

**Microbes as an accomplice.** Microbes are diverse in metabolisms, and some of them can generate toxic metabolic by-products or greenhouse gases, such as H_2_S or CH_4_. These products could have deteriorated marine environments, directly or indirectly, and exacerbated the mass extinction of metazoans. In this sense, the proliferation of microbes would have been an accomplice of mass extinctions.

One role of the proliferation of microbes as an accomplice is, through the decomposition of excess primary production, promoting the formation of widespread anoxic as well as euxinic water columns (anoxic water with free H_2_S). It has been demonstrated that the increased microbial respiration accompanying temperature rise can enhance nutrient cycling and cause intensified marine anoxia [6]. In sulfate-rich marine environments (compared with most freshwater environments), intensified marine anoxia can trigger microbial sulfate-reduction (MSR), which is mainly constrained in pore water of sediments in oxic oceans. MSR is the primary microbial process accounting for the generation of H_2_S. The generated H_2_S, which is usually trapped by reduced metal ions (e.g. Fe^2+^) or re-oxidized to sulfate by O_2_ or other oxidants, can be built up in the anoxia water column to several hundred micromoles, like the Black Sea. Investigations have shown that many mass extinctions were accompanied by intensified MSR in the water column, and in some cases, the euxinic water extended into the photic zone [7].

On the one hand, the buildup of H_2_S would have had direct and substantial impacts on multicellular organisms. For example, most animals could be killed within an hour at an H_2_S concentration >600 ppm. On the other hand, the buildup of H_2_S would have affected seawater chemistry, indirectly impacting the biosphere. In euxinic environments, the concentration of sulfophilic elements such as Mo, Zn and Cu could have decreased significantly. These metals are principal coenzyme factors of critical metabolic enzymes, the depletion of which can substantially weaken the efficiency of the corresponding metabolisms. For example, the depletion of Mo can influence the activity of nitrogenase, the primary enzyme for microbial nitrogen fixation. Furthermore, the depletion of Cu prohibits the function of N_2_O reductase, which is the crucial enzyme for reducing N_2_O to N_2_. Thus, the N_2_O flux to the atmosphere would increase significantly with the presence of widespread oceanic euxinia. Since N_2_O is a vital greenhouse gas whose heat-trapping efficiency is 300 times higher than that of CO_2_, the elevated flux of N_2_O would have created a positive feedback loop in terms of global warming [8].

**Microbes as a savior.** As mentioned earlier, microbes play a substantial role in shaping the habitability of our Earth. For example, the arrival of microbial nitrogen fixation supplied an amount of biologically available nitrogen to the biosphere, which overcame the limitation of nutrient nitrogen, and the O_2_ produced by oxygenic photosynthesis paved the way for the origin of the Eukaryote. Could it be possible that the proliferation of microbes was a savior when it came to mass extinctions?

One potential savior is the microbial mat, which may have provided a refuge for metazoans in widespread-anoxia environments. Mass extinctions in the Phanerozoic (e.g. the F-F and the P-Tr mass extinctions) were accompanied by widespread oceanic anoxia, which is hostile to metazoans [7]. Because of oxygenic photosynthesis by cyanobacteria, the O_2_ concentration in the top several millimeters of modern microbial mats can be four times higher than the overlying water column [9]. These O_2_-rich layers are supposed to have been the main habitats of the early animals in the Late Neoproterozoic [9] and likely have served as an essential oxic oasis for animals that survived through mass extinctions. Some clues from the distribution of ostracods during the P-Tr mass extinction support this hypothesis [10].

The second example of microbes as a savior is carbon fixation, which could have had a negative impact on global warming. Cyanobacteria are the primary producers on modern Earth and could be responsible for ∼25% of net primary productivity in the ocean [11]. Multiple observations, including widespread microbialites and high values of 2α-methylhopane index coupled with low nitrogen isotope compositions, reveal that cyanobacteria generally flourished during mass extinctions [4,5,12]. In widespread-anoxia oceans, the flux of phosphorus recycled diagenetically back to seawater could increase vastly. Since cyanobacteria are able to fix atmospheric N_2_, the elevated phosphorus concentration could promote the proliferation of cyanobacteria and substantially increase oceanic primary productivity. In addition, it has been shown that the metabolic rate and abundance of cyanobacteria can increase significantly in the case of temperature rise [11]. Therefore, it is likely that the proliferation of cyanobacteria played an important role in carbon fixation and in impeding the temperature rise that occurred during many mass extinctions.

The third potential role of microbes as a savior is in the removal of toxic substrates. As discussed above, the buildup of H_2_S in the water column has substantial impacts on the metazoan community and biogeochemical cycles. It is interesting to note that some microbes can oxidize H_2_S and simultaneously fix CO_2_. One type belongs to anoxygenic photosynthesis, e.g. green sulfur bacteria, which have been observed accompanying several mass extinctions, including the F-F, P-Tr and end Triassic [6]. The other type belongs to the chemoautotrophs, e.g. *Thioploca* and *Beggiatoa*, which oxidize H_2_S by O_2_ or nitrate. These microbes have been widely observed in the upwelling area off the eastern Pacific, where euxinic environments are present in the oxygen minimum zone, and may help to impede the extension of H_2_S into shallower water. Although these microbes have not been reported in deep time, they likely thrived and played similar roles during mass extinctions.

**Future directions.** It is evident that microbes interact with the environment in complex ways. They were much involved in mass extinctions, and their environmental feedback was twofold—positive for some macroorganisms and negative for others. Could some of the frequent variations in geological and geochemical records, such as the variations of δ^13^C (carbon isotope composition) observed in the Early Triassic, be caused by the seesaw effect of this positive and negative feedback? In order to quantify the dual role of microbes in environmental change, it is important to couple microbial ecosystem models to Earth system models, e.g. the community earth system model (CESM). For example, the preliminary modules of plankton ecology and microbial processes of S-Fe have been incorporated into the carbon-centric grid-enabled integrated Earth system model (cGEnIE) [13,14]. In such modeling, microbial functional groups closely related to the physicochemical parameters are the principal elements. Firstly, research has to quantify the physicochemical-condition-dependent metabolic parameters of principal microbial pathways precisely. Secondly, the coupled interactions between different microbial functional groups have also to be quantified, which may provide insight into the cryptic geochemical cycles that are usually omitted in geochemical cycle modeling. Notably, as a special form of microbial life, viruses play a substantial role in the biogeochemical cycle of carbon, nitrogen and sulfur. However, the interactions between viruses and other organisms and their environmental consequences are generally omitted in the study of deep-time biogeochemical cycles [15]. Thirdly, proxies that can more precisely quantify the variations in physicochemical conditions are also urgently required.

## Supplementary Material

nwad291_Supplemental_File
