# Supplementary material for: Microbes in mass extinction: an accomplice or a savior?
Source: Natl Sci Rev. 2023 Nov 21;11(1):nwad291. doi: 10.1093/nsr/nwad291 (PMC10727842; doi:10.1093/nsr/nwad291)
Supplement: nwad291_Supplemental_File [file nwad291_supplemental_file.docx]

Supplementary Materials for

**Microbes in mass extinction: An accomplice or a savior**

Genming Luo*, Deng Liu, Hao Yang

State Key Laboratory of Geobiology and Environmental Geology, China University of Geosciences, Wuhan 430074, China

*Corresponding author. Email: gmluo@cug.edu.cn

**This file includes the detailed description of Figure 1 in the main text and Figure S1 cited in the main text.**

Figure 1: (a) Temporal variations of species diversity in the Palaeozoic (1) and family diversity in the Phanerozoic (2), and the abundance of microbial carbonate relative to the metazoan reef (3). The red arrows represent the Big 5 mass extinctions in the Phanerozoic. (b-d) The proliferation of microbes unraveled by geological and geochemical records during the end-Ordovician, Late Devonian (including the end-Devonian), P-Tr, and end-Triassic mass extinctions. (b) Diversity and relative abundance of the microbialite-dominated reefs. The data on the relative abundance of microbialite-dominated reefs is from ref. (4), and the types of microbialite are synthesized from ref. (3-4, 5-10). (c) Microbes revealed by lipid biomarkers. Data from ref. (11-23). (d) Microbes inferred from C-N-S isotope compositions. Data from ref. (16, 24-34).


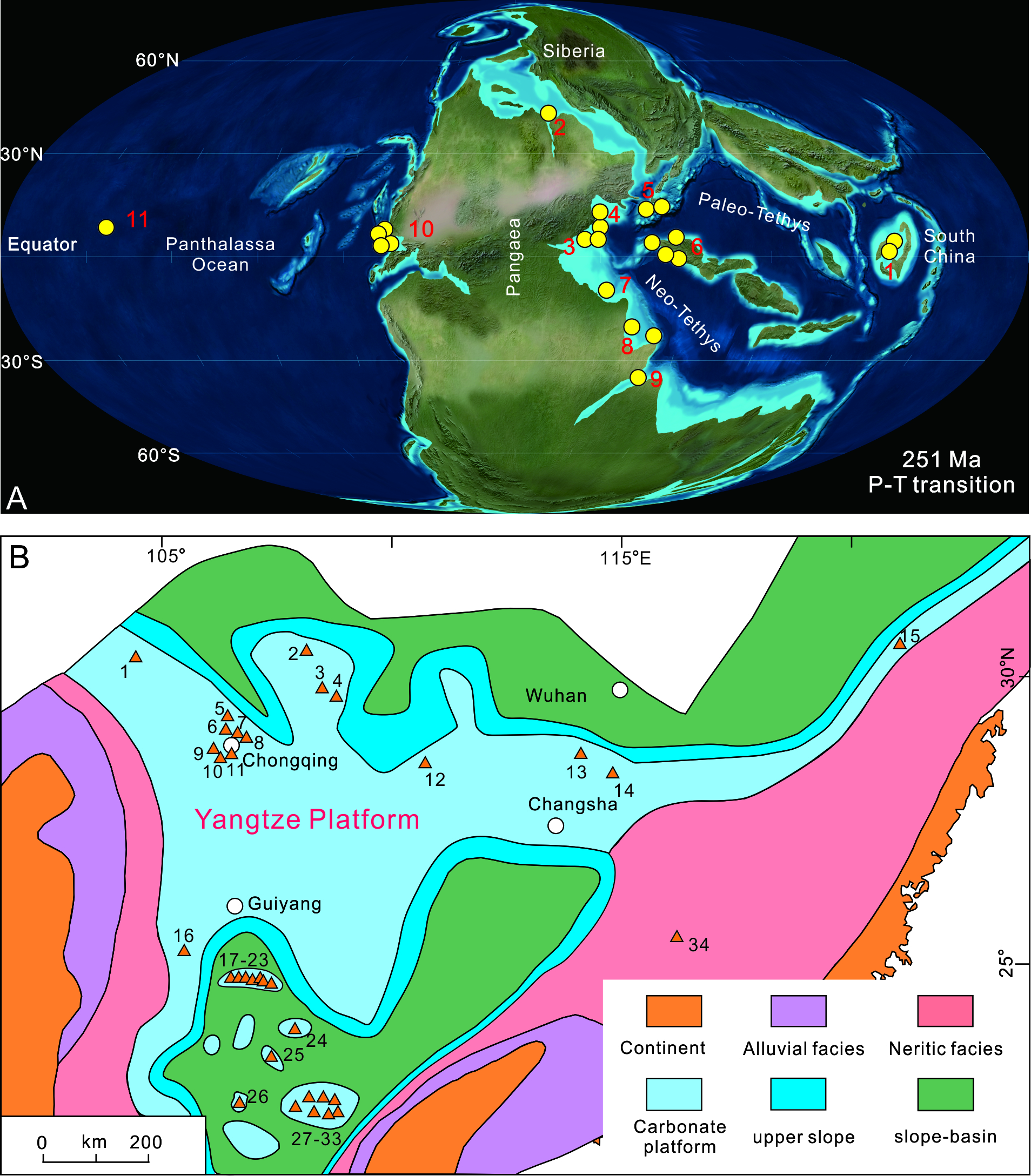


Figure S1 Spatial distribution of microbialites in the Early Triassic after the P-Tr mass extinction. (a) Global distribution of microbialites in the Early Triassic (revised from ref. 35). 1: South China and Vietnam, 2: Greenland, 3: northern Italy and Slovenia, 4: Hungary, 5: Caucasus, 6. Central Iran and Armenia, 7: southern Turkey, 8: southwest Iran, UAE and Oman, 9: Madagascar, 10: Nevada and Utah (USA), 11: southern Japan. (b) Sections cropping out in South China that contain microbialites above the P-Tr mass extinction (revised from ref. 3, 36). Section number codes: 1: Yudongzi, 2: Panlongdong, 3: Xiejiacao-1, 4: Jianshuigou, 5: Baizhuyuan, 6: Dongwan, 7: Tudiya, 8: Laolongdong, 9: Wenxinchang, 10: Xiejiacao, 11: Ruiping, 12: Cili, 13: Chongyang, 14: Xiushui, 15: Xishan, 16: Ziyun, 17-23: Rongbao, Laibin, Dajiang, Heping, Dongjiawan, Xinbaihou, Hochang, 24: Jinya, 25: Tianwan, 26: Zuodeng, 27-33: Longling, Leye, Lingyun, Taiping, Fengshan, Donglan, Pingguo, 34: Tieshikou.

**References**

1. Fan J, Shen S and Erwin DH, *et al*. A high-resolution summary of Cambrian to Early Triassic marine invertebrate biodiversity. *Science* 2020; **367**: 272-277.

2. Raup DM and Sepkoski JJ. Mass extinctions in the marine fossil record. *Science* 1982; **215**: 1501-1503.

3. Chen Z, Tu C and Pei Y, *et al*. Biosedimentological features of major microbe-metazoan transitions (MMTs) from Precambrian to Cenozoic. *Earth-Sci. Rev.* 2019; **189**: 21-50

4. Mata SA and Bottjer DJ. Biosedimentological features of major microbe-metazoan transitions (MMTs) from Precambrian to Cenozoic. *Geobiology* 2012; **10**: 1-22.

5. Sheehan PM and Harris MT. Microbialite resurgence after the Late Ordovician extinction. *Nature* 2004; **430**: 75-78.

6. Kershaw S, Crasquin S and Li Y *et al*. Microbialites and global environmental change across the Permian–Triassic boundary: a synthesis. *Geobiology* 2011; **10**: 25-47.

7. Yang H, Chen Z and Wang YB *et al*. Composition and structure of microbialite ecosystems following the end-Permian mass extinction in South China. *Palaeogeogr. Palaeoclimatol. Palaeoecol.* 2011; **308**: 111-128.

8. Ibarra Y, Corsetti FA and Greene SE *et al*. A microbial carbonate response in synchrony with the end-Triassic mass extinction across the SW UK. *Sci. Rep.* 2016; **6**: 19808.

9. Peterffy O, Calner M and Vajda V. Early Jurassic microbial mats—A potential response to reduced biotic activity in the aftermath of the end-Triassic mass extinction event. *Palaeogeogr. Palaeoclimatol. Palaeoecol.* 2016; **464**: 76-85.

10. Yao L, Aretz M and Chen J *et al*. Global microbial carbonate proliferation after the end-Devonian mass extinction: Mainly controlled by demise of skeletal bioconstructors. *Sci. Rep.* 2016; **6**: 39694.

11. Xie SC, Pancost RD and Yin HF *et al*. Two episodes of microbial change coupled with Permo/Triassic faunal mass extinction. *Nature* 2005; **434**: 494-497.

12. Xie SC, Algeo TJ and Zhou WF *et al*. Contrasting microbial community changes during mass extinctions at the Middle/Late Permian and Permian/Triassic boundaries. *Earth Planet. Sci. Lett.* 2017; **460**: 180-191.

13. Grice K, Cao CQ and Love GD *et al*. Photic zone euxinia during the Permian-Triassic superanoxic event. *Science* 2005; **307**: 706-709.

14. Grice K, Twitchett RJ and Alexander R *et al*. A potential biomarker for the Permian-Triassic ecological crisis. *Earth Planet. Sci. Lett.* 2005; **236**: 315-321.

15. Schwark L and Empt P. Sterane biomarkers as indicators of palaeozoic algal evolution and extinction events. *Palaeogeogr. Palaeoclimatol. Palaeoecol.* 2006; **240**: 225-236.

16. Cao CQ, Love GD and Summons RE *et al*. Biogeochemical evidence for euxinic oceans and ecological disturbance presaging the end-Permian mass extinction event. *Earth Planet. Sci. Lett.* 2009; **281**: 188-201.

17. Jia CL, Huang JH and Kershaw S *et al*. Microbial response to limited nutrients in shallow water immediately after the end-Permian mass extinction. *Geobiology* 2012; **10**: 60-71.

18. Marynowski L, Zatoń M and Rakociński M *et al*. Deciphering the upper Famennian Hangenberg Black Shale depositional environments based on multi-proxy record. *Palaeogeogr. Palaeoclimatol. Palaeoecol.* 2012; **346–347**: 66-86.

19. Luo GM, Wang YB and Grice K *et al*. Microbial–algal community changes during the latest Permian ecological crisis: Evidence from lipid biomarkers at Cili, South China. *Global Planet. Change* 2013; **105**: 36-51.

20. Rohrssen M, Love GD and Fischer W *et al*. Lipid biomarkers record fundamental changes in the microbial community structure of tropical seas during the Late Ordovician Hirnantian glaciation. *Geology* 2013; **41**: 127-130.

21. Kasprak AH, Sepúlveda J and Price-Waldman R *et al*. Episodic photic zone euxinia in the northeastern Panthalassic Ocean during the end-Triassic extinction. *Geology* 2015; **43**: 307-310.

22. Whiteside JH and Grice K. Biomarker records associated with mass extinction events. *Annu Rev Earth Planet Sci.* 2016; **44**: 581-612.

23. Smolarek J, Marynowski L and Trela W *et al*. Redox conditions and marine microbial community changes during the end-Ordovician mass extinction event. *Global Planet. Change* 2017; **149**: 105-122.

24. Zhang TG, Shen YA and Zhan R *et al*. Large perturbations of the carbon and sulfur cycle associated with the Late Ordovician mass extinction in South China. *Geology* 2009; **37**: 299-302.

25. Luo GM, Kump LR and Wang YB *et al*. Isotopic evidence for an anomalously low oceanic sulphate concentration following end-Permian mass extinction. *Earth Planet. Sci. Lett.* 2010; **300**: 101-111.

26. Luo GM, Wang YB and Algeo TJ *et al*. Enhanced nitrogen fixation in the immediate aftermath of the latest Permian marine mass extinction. *Geology* 2011; **39**: 647-650.

27. Luo GM, Xie SC and Liu D *et al*. Microbial influences on paleoenvironmental changes during the Permian-Triassic boundary crisis. *Sci. China Earth Sci.* 2014; **57**: 965-975.

28. Luo GM, Algeo TJ and Zhan R *et al*. Perturbation of the marine nitrogen cycle during the Late Ordovician glaciation and mass extinction. *Palaeogeogr. Palaeoclimatol. Palaeoecol.* 2016; **448**: 339-348.

29. Luo GM, Richoz S and van de Schootbrugge B *et al*. Multiple sulfur-isotopic evidence for a shallowly stratified ocean following the Triassic-Jurassic boundary mass extinction. *Geochim. Cosmochim. Acta* 2018; **231**: 73-87.

30. Melchin MJ, Mitchell CE and Holmden C *et al*. Environmental changes in the Late Ordovician–early Silurian: Review and new insights from black shales and nitrogen isotopes. *GSA Bull.* 2013; **125**: 1635-1670.

31. Liu JS, Qie WK and Algeo TJ *et al*. Changes in marine nitrogen fixation and denitrification rates during the end-Devonian mass extinction. *Palaeogeogr. Palaeoclimatol. Palaeoecol.* 2016; **448**: 195-206.

32. Schoepfer SD, Algeo TJ and Ward PD *et al*. Testing the limits in a greenhouse ocean: Did low nitrogen availability limit marine productivity during the end-Triassic mass extinction? *Earth Planet. Sci. Lett.* 2016; **451**: 138-148.

33. He T, Dal Corso J and Newton RJ *et al*. An enormous sulfur isotope excursion indicates marine anoxia during the end-Triassic mass extinction. *Sci. Adv.* 2020; **6**: eabb6704.

34. Wu BJ, Luo GM and Joachimski MM *et al*. Carbon and nitrogen isotope evidence for widespread presence of anoxic intermediate waters before and during the Permian-Triassic mass extinction. *GSA Bull.* 2022; **134**: 1397-1413.

35. Foster WJ, Heindel K and Richoz S *et al*. Suppressed competitive exclusion enabled the proliferation of Permian/Triassic boundary microbialites. *Depos. Rec.* 2020; **6**: 62-74.

36. Yin HF, Jiang HS and Xia WC *et al*. The end-Permian regression in South China and its implication on mass extinction. *Earth-Sci. Rev.* 2014; **137**: 19-33.
